# Clinical value of DPOC for detecting and removing residual common bile duct stones (video)

**DOI:** 10.1186/s12876-019-1045-6

**Published:** 2019-07-26

**Authors:** Jun-Jie Yang, Xiong-Chang Liu, Xiao-Qin Chen, Qi-Yong Zhang, Tian-Rang Liu

**Affiliations:** Department of Gastroenterology, Lanzhou First People’s Hospital, No. 1 of Wujiayuan west Street, Qilihe District, Lanzhou City, 730050 China

**Keywords:** Common bile duct stones (CBDs), Direct peroralcholangioscopy (DPOC), Endoscopic retrograde cholangiopancreatography (ERCP), Cholangiography, Residual stones

## Abstract

**Background:**

This study aims to evaluate the efficacy and safety of detecting and removing residual common bile duct stones (CBDS) using direct peroralcholangioscopy (DPOC) after performing endoscopic retrograde cholangiopancreatography (ERCP) for stone retrieval.

**Methods:**

From January 5, 2017 to December 27, 2017, a total of 164 cases of choledocholithiasis were treated by ERCP for stone retrieval. According to the inclusion and exclusion criteria, the remaining 79 cases (39 males; mean age: 63.3 years old, range: 52–79 years old) were enrolled in the present study. The maximum transverse stone diameter was 6–15 mm (12.7 ± 4.2 mm), as determined by ERCP. Furthermore, there were 57 cases of multiple stones (number of stones: two in 41 cases, three in nine cases, and ≥ 4 in seven cases), 13 cases of post-mechanical lithotripsy, and nine cases of broken stones.

**Results:**

The overall success rate of DPOC was 94.9% (75/79). Furthermore, 18.7%(14/75) of cases were directly inserted, 72%(54/75) of cases required guide wire assistance, and 9.3%(7/75) of cases were successfully inserted with overtube assistance. The average insertion time was 7–17 min (4.9 ± 2.9 min). Residual stones were detected in 19 cases (25.3%), and all of which were < 5 mm in diameter. Moreover, five cases of formed stones were removed by basket and balloon catheter, while the remaining cases were cleaned after irrigation and suction. There were no serious complications.

**Conclusion:**

DPOC is safe and effective for both the detection and removal of residual CBDS after conventional ERCP.

**Electronic supplementary material:**

The online version of this article (10.1186/s12876-019-1045-6) contains supplementary material, which is available to authorized users.

## Background

Endoscopic sphincterotomy (EST) and/or endoscopic papillary balloon dilatation (EPBD) have become the first choice for the treatment of choledocholithiasis [1]. Cholangiography is generally performed to confirm bile duct clearance after stone retrieval. However, cholangiography may be an imperfect tool for this diagnostic purpose. Small stones may be overlooked due to concealment by contrast agents [2], which may increase the risk of recurrence of stones in the future [3]. These residual bile duct stones can be identified by intraductal ultrasonography (IDUS) and choledochoscopy, but several disadvantages limit the application of this approach [4]. At present, there are reports on the application of direct peroralcholangioscopy (DPOC) for difficult common bile duct stones (CBDSs) [5–7]. This was applied by the investigators for the diagnosis and treatment of residual stones after routine methods. The results are summarized as follows.

## Materials and methods

### Patients

From January 5, 2017 to December 27, 2017, a total of 164 cases of choledocholithiasis were treated by endoscopic retrograde cholangiopancreatography (ERCP) for stone retrieval. All patients were prepped for abdominal ultrasound and magnetic resonance cholangiopancreatography (MRCP) before the operation to determine the size, number and location of the stones. Inclusion criteria: broken stones during routine stone removal, repeated stone removal with multiple stones, or mechanical lithotripsy with difficult stones. Exclusion criteria: cholecystolithiasis, a common bile duct diameter of< 10 mm, the complete removal of single stones, or inability to tolerate ERCP due to a combination of severe systemic diseases. The remaining 79 cases (39 males; mean age: 63.3 years old, range: 52–79 years old) were enrolled in the present study and underwent DPOC to determine whether there were any stone remnants. Among these patients, parapapillary diverticulum was present in 24 cases (30.4%), recurrent stones occurred in 29 cases (36.7%), and prior cholecystectomies occurred in 17 cases (21.5%). The maximum transverse stone diameter was 6–15 mm (12.7 ± 4.2 mm), as determined by ERCP. There were 57 cases of multiple stones (number of stones: two in 41 cases, three in nine cases, and ≥ 4 in seven cases), 13 cases of stones at post-mechanical lithotripsy, and nine cases of broken stones (Table 1).

**Table 1:** Patients characteristics [n (%)]

Characteristics			*n* = 79
Age (yr)			63.3 ± 10.5 (range, 52–79)
Gender (male)			39 (49.4)
Concurrent Diseases	Prior cholecystectomies		17 (21.5)
	Parapapillary diverticulum		24 (30.4)
	Patients with recurrent cbd stones		29 (36.7)
Operation Cause	Multiple stones	2	41
		3	9
		≥4	7
	Post-mechanical lithotripsy		13
	Broken stones		9

### Instruments

An ultraslim endoscope and duodenoscope (EG530N and ED530XT; Fujinon Corporation, Omiya, Japan), high-frequency electrocautery (VIO300S; ERBE Elektromedizin, Tubingen, Germany), guidewire (Turumo [China] Holdings Co.Ltd., Hangzhou, China), Jagwire (Boston Scientific, Natick, MA, USA), pull-type sphincterotome (ENDO-FLEX GmbH; Voerde, Germany), dilation balloon (Microvasive; Boston Scientific), extractor balloon and extractor basket (Cook Medical Co., Winston-Salem, NC, USA), extractor balloon and extractor basket specially designed for ultraslim endoscope (Changzhou Jiuhong Medical Instrument Co. Ltd. Changzhou, China), and overtube dedicated for ultraslim endoscope (Patent number: ZL2013 206631516) were used.

### Methods

Anesthesia and preoperative preparation were performed prior to ERCP. Antibiotics were given at 30 min before the operation, and at 8–10 h and 48 h after the operation. All patients were monitored by anesthesiologists while under propofol plus fentanyl intravenous anesthesia.

The size, number and position of the calculi were determined by ERCP. In the 54 patients who underwent small EST, EPBD was used with 10–15 mm balloon dilatation (The diameter of the balloon is the maximum diameter of the stone plus 2 mm, and the maximum diameter was < 15 mm). Furthermore, 22 patients underwent EST only because the papilla shapes were very suitable for incision. Merely three patients underwent EPBD due to the papilla in the diverticulum. The basket/balloon was removed to remove the stones, and radiography confirmed that no stone shadow was present following the EST or/and EPBD.

### Endoscopic insertion

#### Direct insertion method

An ultraslim endoscope was inserted through the mouth into the descending segment of the duodenum, turned right under X-ray surveillance, and the ultraslim endoscope was straightened. When the endoscopic tip was located below the duodenal papilla, the tip was turned upwards and pulled back continuously. Hence, the ultraslim endoscope entered the lower part of the common bile duct through the enlarged papillary opening as the colonoscope entered the ileum through the ileocecal valve (Additional file 1). Next, the ultraslim endoscope was repeatedly rotated and manipulated to insert it into the target position or the hepatic hilum.

#### Guidewire guiding method

**Fig. 1:**
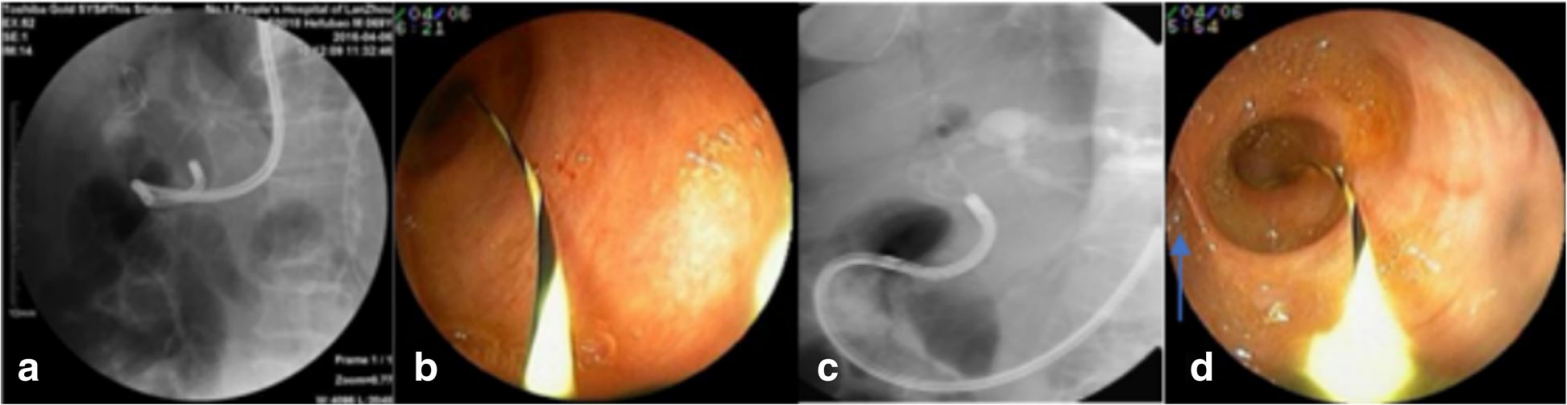
Guide wire guide method. **a** ultraslim endoscope through the opening of the duodenal papilla to the distal end of the bile duct; **b** insertion guide wire through the endoscopic working channel; **c** fluoroscopy confirmed that the guide wire reached the intrahepatic bile duct; **d** ultraslim endoscope insertion to the common hepatic duct along the guide wire, arrow pointed to the opening of the cystic duct

This method was suitable for use in the instance of direct insertion failure. The ultraslim endoscope was aligned to the opening of the papilla (Fig. 1a), the J-shaped guide wire was inserted into the proximal bile duct or hilar (Fig. 1b and c), and the ultraslim endoscope was inserted along the guide wire into the bile duct target position (Fig. 1d).

#### Overtube assistance method

**Fig. 2:**
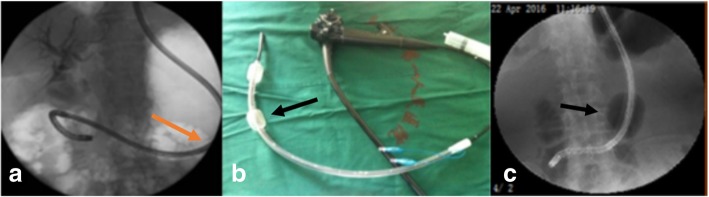
Overtube assistance method. **a** ultraslim endoscope reached the descending portion of the duodenum but unable to enter the common bile duct because it coiled in the greater curvature of the stomach (yellow arrow); **b** overtube was installed in vitro, black arrow show the oral balloon; **c** ultraslim endoscope and overture were inserted again, the balloon was inflated after the ultraslim endoscope entered the duodenum, then the ultraslim endoscope was straightened

If the ultraslim endoscope coils in the stomach or duodenum with repeated failure attempted to resolve the situation (Fig. 2a), the ultraslim endoscope was withdrawn and re-inserted after installation of an auxiliary overtube (Fig. 2b). When it reached the descending duodenum, it was pulled back and turned right to the ultraslim endoscope. Next, the overtube was inserted along the ultraslim endoscope to the proximal balloon across the cardia (on a 43 cm scale), and the balloon was inflated (Fig. 2c). Then, an ultraslim endoscope was inserted along the cannula to reduce the intragastric loop until entry.

### Removal of the stone

Once the ultraslim endoscope was inserted into the common bile duct, the residual stones would be usually located at the distal end of the bile duct or front end of the ultraslim endoscope by attraction, which could easily to be found. For the forming stone, the stone could be caught and taken out by the basket or balloon catheter. For the paste stone, small/numerous paste-like stones could be cleaned after irrigation and suction.

### Postoperative treatment

This was the same as conventional ERCP.

## Results

The ultraslim endoscope was able to reach the hepatic hilum in 75 patients (94.9%). Among these patients, it was inserted directly in 14 patients (18.7%), it required guidewire assistance in 54 patients (72%), and it was successfully inserted with overtube assistance in seven patients (9.3%). For the remaining four patients, the bile duct was extended due to having cholecystectomy, while this failed to be inserted under fluoroscopy. The insertion time of ulthaslim endoscope from mouth to common bile duct or hepatic hilar for these 75 successful operations ranged within7–17 min (average: 4.9 ± 2.9 min). The relationship of DPOC between the insertion method and success rates are shown in Table 2.

**Table 2:** Success rates and inserting method of endoscopic insertion

	Overall success rate	Failure rate	Direct insertion	Guide wire assistance	Overtube assistance
Success rate	94.9 (75/79)	5.1 (4/79)	18.7 (14/75)	72 (54/75)	8.9 (7/75)

Residual stones were detected in 19 patients (25.3%), in which 24.5% (13/53) were due to multiple stones, 23.8% (5/21) were due to post-mechanical lithotripsy, and 11.1%(1/9) were due to broken stones. All stones were < 5 mm in diameter. The number of residual stones was one in seven patients, two in two patients, and three in three patients, while in seven patients, the stones were in a paste. Furthermore, stones in five cases were removed by basket and balloon catheter, while the rest were cleaned after irrigation and suction.

There were no serious complications, such as bleeding, perforation, or severe pancreatitis, and there were no operation-related deaths. The total complications rate was 6.7%(5/75), in which three patients (4.0%) had postoperative fever, right upper abdominal pain, increased white blood cells, and elevated calcitonin. These patients were presumed to be evidenced with a biliary tract infection, and improved by using antibiotics. Postoperative pancreatitis occurred in two patients (2.7%, mild in one patient and moderate in the other patient, respectively).

## Discussion

**Fig. 3:**
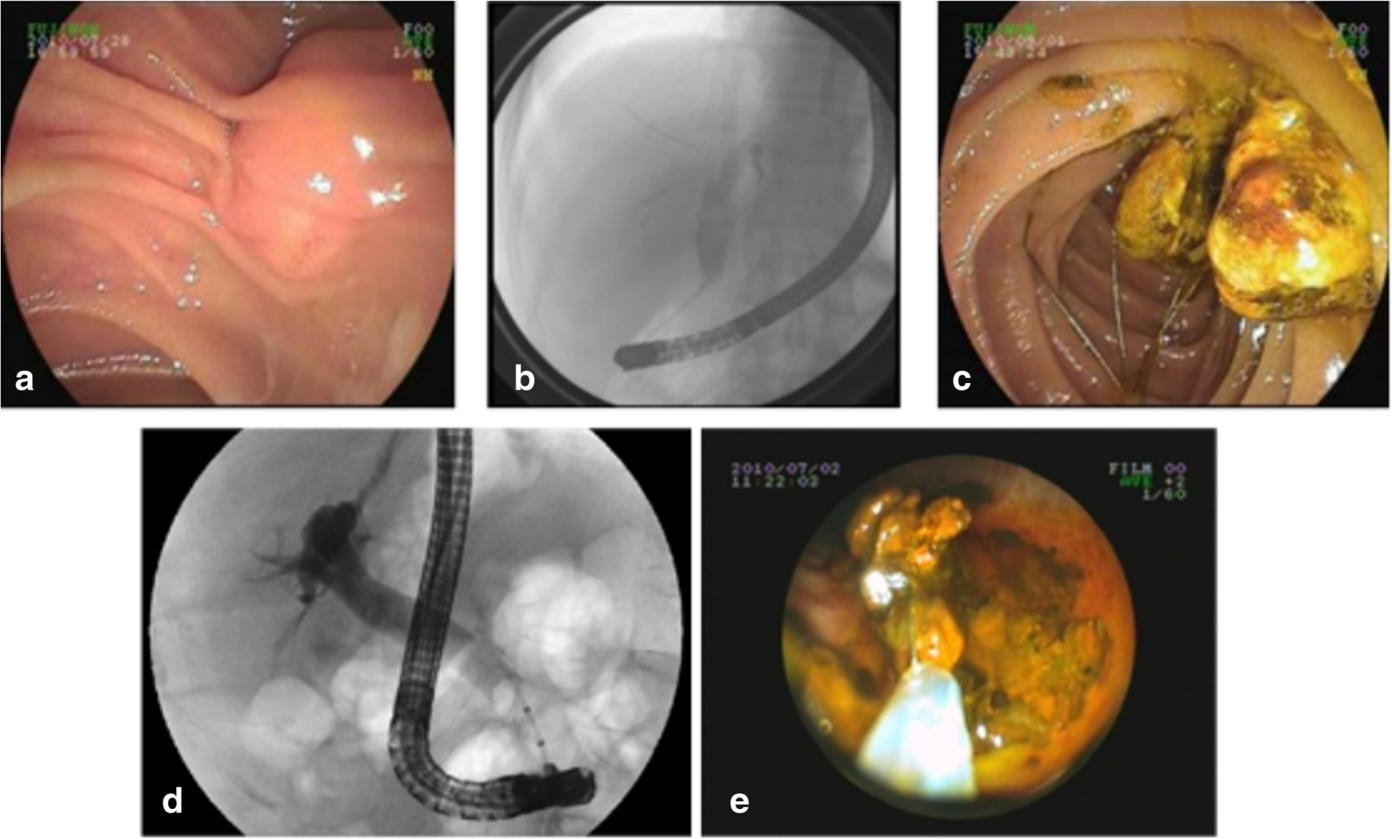
Residual stones were detected by DPOC after mechanical lithotripsy. **a** well-conditioned duodenal papilla; **b** 1.8 × 2.5 cm filling defect detected by choledochography; **c** removal of broken stones after EST plus EPBD and mechanical lithotripsy; **d** no cholangiographic filling defect were found after balloon cleaning; e:more common bile duct residual stones were found and clean up by DPOC

Conventional ERCP combined with EST/EPBD can achieve a success rate of approximately 90% [8]. It remains to be determined whether conventional ERCP stone extraction can completely clean up these stones, and this has been poorly researched at present. Furthermore, cholangiography is generally performed to confirm bile duct clearance after stone removal [4, 9]. However, this method is not completely accurate. Studies have shown that small stones or fragments may be not found, because these are obscured by contrast agents [9], especially after repeated injections of contrast agents due to multiple removal operations or the lithotripsy of larger stones (Fig. 3a, b, c and d). Huang et al. reported a residual stone rate of 22.7% [10]. Similarly, Itoi [5] identified a residual stone rate of 24% (26/108) by mother-baby cholangioscopy. Both studies included patients with cholecystolithiasis. The investigators found that residual stones mainly occurred in multiple stones (24.5%) and post-mechanical lithotripsy (23.8%). In order to rule out the possibility of gallbladder stones falling into the bile duct as the papillary sphincter pressure dropped, patients without gallbladder stones or post-cholecystectomy were analyzed. The residual stone rate was similar to these studies, suggesting that these residual stones were missed during the ERCP, and did not result from gallbladder stones migrating into the bile duct.

In addition to cholangiography, intraductal ultrasound (IDUS) and cholangioscopy are two methods that can help in the diagnosis of residual CBDSs. Tsuchiya [11] found that by using IDUS, stone residue was observed in 23.7% (14/59) of patients. However, ultrasound probes are expensive, easily damaged, provide poor image quality, and have high technical dependence. These limit the application of this technique for the diagnosis of residual stones. Furthermore, there are risks for overlooked residual stones. Ohashi [4] determined that the rate of inaccurate detection of residual stones by IDUS was 14.6% (6/41). SpyGlass or mother-baby cholangioscopy are also not suitable for the diagnosis and treatment of residual CBDSs due technical limitations, and the 1.2 mm working channels were not capable of removing these stones. DPOC allows for a wide range of endoscopic sources and good image quality. Since residual stones are often located at the distal end of the common bile duct, it can be clearly determined whether any residual stone is present once ultraslim endoscope passes through the papillary opening into the bile duct.

Several ultraslim or transnasal video endoscopes are commercially available [12]. These scopes have a four-way angulation function and outer diameters of 5.0–5.9 mm, with a 2-mm working channel, providing excellent images. Furthermore, these have an image-enhanced function system. There are already more advanced virtual 3D-cholangioscopious applications for clinical research [13]. DPOC can be used only in dilated bile ducts due to the larger diameter. Hence, EST and/or balloon dilation should be mandatory as a pretreatment for its smooth insertion into the bile duct through the papilla. However, there are obstacles that need to be overcome with this technique. The biggest difficulty is the low success rate of endoscopic insertion. Due to the presence of stomach curvature, it is impossible to move the ultraslim endoscope freely during the operation. It has been reported in a literature that the success rate of intubation was < 50% when certain assistant tools were not used [14]. Some techniques of scope insertion have been reported [15–17], as follows: (1) direct scope insertion without any devices, (2) wire-guided insertion, (3) overtube balloon-assisted insertion, (4) duodenal balloon-assisted insertion, and (5) intraductal balloon catheter-assisted insertion. However, it remains unclear which technique is better [12]. Moon’s study revealed that the success rate of intubation guided by a balloon was much higher than that guided by a wire (95.2% vs. 45.4%) [14]. A combination of methods can increase the success rate of insertion from 45.5 to 95% [18]. However, since the anchoring of the balloon leads to serious complications, such as gas embolism, the manufacturer has withdrawn from the market [19]. Regardless of whether the ultra-stiff guidewire or anchoring balloon catheter is used, it is extremely difficult to support the ultraslim endoscope, and it cannot prevent the ultraslim endoscope from bending into the large curvature of the stomach. Although the overtube can prevent the formation of loop in the stomach, this technique is presently used in enteronoscopy, which is too thick and hard for an ultraslim endoscope. In the present study, 91.1% success rate was obtained using the direct insertion method or ordinary guidewire, and the operation was simple and the time was short. The key point was that the papillary orifice must be fully expanded to make the ultraslim endoscope pass smoothly, and reduce the possibility of intergastric loops. Since the commonly used ultraslim endoscope has a diameter of 5–6 mm, EST and/or EPBD is required, but incision or expanding too much increases the probability of bleeding, perforation and postoperative pancreatitis. The diameter of the bile duct in the present study was all above 10 mm. Although some of the cases of ampulla were in or next to the diverticulum, there was space for a small incision (Fig. 1). Therefore, the small incision and balloon expansion technique (at least 10 mm) far exceeds the outer diameter of the ultraslim endoscope, ensuring that the ultraslim endoscope can successfully cross the orifice of the major papilla into the bile duct. Furthermore, it also reduces complications. In order to reduce the formation of a loop for the ultraslim endoscope in the stomach or duodenum, when the ultraslim endoscope reaches the duodenal descending segment, the ultraslim endoscope is turned right and pulled back to straighten it. Then, it is turned left and continuously pulled. The ultraslim endoscope can enter the distal segment of the CBD through the enlarged papillary opening in the same way as the colonoscopy enters the ileum through the cecum. In some cases, the bile duct bends to the upper left, and often needs to be inserted into the hepatic hilar with the guidance of a wire guide. An overtube can be used for individually repeated solution loop loser. In four patients the operation failed as the choledochus became tortuous and extended after cholecystectomy, thereby it was difficult to deeply insert or observe the hilus, although it successfully passed through the papillary opening. Direct insertion method or guide wire assistant method is easy to operate and saves time, combined with overtube assistant method for the difficult patients can make the overall insertion success rate reach 94.9%. At present, there are various new technologies to improve the success rate of DPOC [12, 20–23].

Previous studies on both IDUS and cholangioscopy [24–26] have been consistent with the findings of the present study, confirming that retained stones are often < 5 mm in diameters, the opening after incision is large enough, and therefore, self-drainage is possible. However, it remains unclear as to whether these small residual stones are of clinical significance. Itoi [5] found that 24% of stones remained in the biliary in the examination conducted at six days after quarrying, suggesting that these stones may persist in the long-term, and eventually lead to stone recurrence. A number of studies have aimed at analyzing the risk factors for the recurrence of stones, and suggested that stone residue is a possible cause of recurrence [2, 3]. Tsuchiya [11] reported that IDUS can reduce the recurrence rate of stones from 13.2 to 3.4%. Therefore, it is possible to find and remove residual stones, in order to reduce the risk of stone recurrence. However, long-term follow-up results are needed to confirm these findings in larger populations.

In the present study, since the ultraslim endoscope in the 2-mm working channel could pass through the 5Fr balloon catheter or basket catheter, and since these residual stones were often small, stone clearance was relatively simple (Fig. 3e). Formed stones could be directly examined in the basket or balloon catheter. Paste residue was removed via endoscopic irrigation and suction, combined with the balloon catheter (Additional file 2). Fugazzaet al. [27] reported 20 patients with difficult biliary stones, who underwent DPOC to verify the complete clearance of CBD stones. The intubation and guidewire assistance success rate, mean investigation time, and incidence of complications were similar to the present study.


**Additional file 2:** Endosopic basket removal of residual stones (MP4 10279 kb)


The present study demonstrates that DPOC is a safe technique in the described format. Furthermore, the incidence of complications was low, with the most common being postoperative cholangitis. According to one of the largest series of studies published, to date [28], and despite the use of prophylactic antibiotics, the incidence of postoperative cholangitis remains at 10%, which is higher than the rate of 4.2% (4/96) observed in the present study. The reason why the incidence of complications of biliary tract infection in the present study was lower than that reported in the literature is that there was no biliary stricture in all cases, and the papillary opening was sufficiently enlarged to reduce the damage of biliary mucosa caused by endoscopy, and the biliary tract was sufficiently washed after stone removal. Other complications, such as bleeding, perforation and postoperative pancreatitis, were similar in frequency to conventional ERCP. However, vigilance is important, since there are reports of rare serious complications of DPOC [29, 30]. Especially gas embolism, which is caused by gas entering the portal vein or liver vein along the injured bile duct wall when endoscopic blockage of papillary opening and excessive gas injection increased biliary pressure. In order to reduce the risk of this complication, gas injection must be minimized, the expanded papillary opening should be sufficiently large, and the smooth entry and exit of the balloon catcher with a diameter of 10 mm should be the minimum standard. Furthermore, the ultraslim endoscope into the bile duct should maintain a clear field of vision and deep insertion along the guidewire, in order to avoid blind insertion, which could result in bile duct wall damage. In an animal experiment conducted in South Korea, it was shown that over-inflating the balloon could also lead to perforation of the bile duct [31]. The investigators were warned about the need for great caution when conducting similar a research.

## Conclusions

In summary, the present study found that post-ERC cholangiography is not a reliable method for confirming the complete removal of stones. The use of ultraslim endoscopes for DPOC is a tool useful for determining whether post-ERC stones are cleaned, and is substantially more useful for residual stone extraction.

## Additional file

Additional file 1: DPOC operation proces. (MP4 2115 kb)

## Data Availability

The datasets used and/or analysed during the current study are available from the corresponding author on reasonable request.
